# Defining endotypes of bronchopulmonary dysplasia in preterm infants to improve precision-based therapies

**DOI:** 10.1172/jci.insight.193975

**Published:** 2025-10-22

**Authors:** Megha Sharma, Gangaram Akangire, Noah H. Hillman, Winston M. Manimtim, Mark Ivan Attard, Venkatesh Sampath

**Affiliations:** 1 Department of Pediatrics, Division of Neonatology, University of Arkansas for Medical Sciences, Little Rock, Arkansas, USA.; 2 Arkansas Children’s Research Institute, Little Rock, Arkansas, USA.; 3 Department of Pediatrics, Division of Neonatology, Children’s Mercy Hospital, Kansas City, Missouri, USA.; 4 Department of Pediatrics, Division of Neonatology, Saint Louis University, St. Louis, Missouri, USA.; 5 Neonatal Unit, Aberdeen Maternity Hospital, Grampian University Hospitals NHS Trust, Aberdeen, Scotland, United Kingdom.; 6 Neonatal Diseases Research Program, Children’s Mercy Research Institute, Kansas City, Missouri, USA.

## Abstract

Bronchopulmonary dysplasia (BPD) remains a debilitating disease in premature infants. The chronic pathogenesis of BPD with complex prenatal and postnatal programming challenges attempts at precisely defining or treating disease. While existing BPD definitions categorize disease severity, a lack of consideration of disease heterogeneity and endotypes has contributed to the failure of clinical trials to improve BPD outcomes. Recent studies have used advanced lung imaging techniques, echocardiography, and lung function tests to identify airway, parenchymal, and vascular BPD endotypes. These endotypes carry different prognoses and require endotype-specific treatment strategies to optimize infant outcomes. In this Review, we focus on the pathogenic mechanisms that specify individual BPD endotypes and discuss how combining biomarkers, functional studies, and artificial intelligence–based characterization of endotypes can inform precision therapies for BPD.

## Introduction

Bronchopulmonary dysplasia (BPD) remains the most prevalent chronic lung disease of infancy, with more than 12,000 infants diagnosed annually in the United States (1–3). Infants with BPD are at increased risk of death and suffer from long-term pulmonary disability and neurodevelopmental impairments (1, 4–6). In the 50 years since the term BPD was coined, the demographics and pathogenesis of BPD have evolved; it is now considered a heterogenous disease affecting infants born before 30 weeks gestation (1, 2, 5, 7, 8). While modern BPD definitions are better at predicting intermediate-term clinical outcomes, their inability to discern various pathogenic mechanisms and disease subtypes has contributed to the lack of improvements in clinical outcomes (5, 9). The term “endotype” refers to “a subtype of a condition, which is defined by a distinct functional or pathophysiological mechanism” (10, 11). Clinical trials that have attempted to prevent BPD with specific ventilation strategies, saturation targets, antiinflammatory systemic or inhaled steroids, and pulmonary vasodilator therapy have provided only modest benefit, potentially due to a lack of appreciation of BPD heterogeneity and endotypes (12–18). On a promising note, pulmonary functional studies, advanced lung and cardiovascular imaging, biomarker studies, and artificial intelligence–based (AI-based) phenomics approaches appear to identify distinct BPD endotypes that lend themselves to precision therapies (19–25). Herein, we combine a historical perspective of BPD definitions and pathogenesis with clinical, functional, and AI-based approaches to characterize BPD endotypes, highlighting the potential for endotype-based precision therapies.

## BPD definitions over time

Northway et al. coined BPD in 1967 based on radiographic assessments of 32 late preterm infants with respiratory distress syndrome (RDS) who received prolonged artificial ventilation and warm, humidified 80%–100% oxygen for >6 days (26). In addition to identifying risk factors, Northway et al. characterized the 4 temporal stages of BPD from early injury to regenerative changes correlating chest radiography changes with lung pathology. An identical phenotype was described by Hawker et al., who in 1967 described infants with RDS who received mechanical ventilation and high-inspired oxygen supplementation (27). However, the very first description of a chronic lung disease phenotype in preterm infants was in 1960 by Wilson and Mikity (28), who described infants that did not require oxygen or respiratory support in the first days of life but subsequently developed respiratory disease, with microcystic lung changes, and progression to a chronic, emphysematous stage in some. There have been subsequent reports of Wilson and Mikity syndrome, and, while the etiology is unclear, it is attributed to perinatal inflammation/infection in the lung (29) (Table 1).

Improved efforts at limiting oxygen toxicity and mechanical ventilation along with better nutrition improved preterm infant (born before 32 weeks gestation) survival in the 1970s but led to an increase in BPD incidence. In 1978, the NIH convened a workshop to develop standard definitions for BPD that would allow comparison across centers and research studies (30). The *Journal of Pediatrics* dedicated an entire issue in 1979 to manuscripts on BPD pathogenesis, clinical features, animal models, and interventions (30). The definition combined a continued need for oxygen in the first 28 days of life with corroborative clinical and radiographic features (8). In 1988, Sheehan proposed defining BPD based on need for supplemental oxygen at 36 weeks postmenstrual age (PMA), as this predicted pulmonary outcomes better at 2 years of age (31). The 36 weeks PMA cut-off remains a part of BPD definitions.

With the use of surfactant, antenatal steroids, and gentler ventilation strategies, many preterm infants in the 1990s did not develop the classical dysplastic/fibrotic BPD phenotype seen in the 1970–1980s, leading Jobe et al. to coin the term “new BPD,” characterized by alveolar simplification (32). Through the 1990s and 2000s, the complex nature of BPD pathogenesis — encompassing heredity factors, lung inflammation, nutrition, male sex, and fluid overload, in addition to traditional risk-factors, such as ventilation, hyperoxia, and infection — was recognized (7, 32, 33). In 2000, an NIH-organized workshop proposed defining BPD as requirement of a minimum of 28 days of supplemental oxygen, with a continued need for oxygen at 36 weeks PMA (33). Moreover, a stratification of BPD was proposed based on severity, defined as mild, moderate, and severe disease (33). To decrease heterogeneity in oxygen delivery, which affects BPD definition, Walsh et al. incorporated a physiological oxygen challenge at 36 weeks PMA to validate BPD diagnosis, as per the 2001 NIH definition (34). The change in the BPD demographic to infants predominantly born at <29 weeks’ gestation and use of newer modes of noninvasive respiratory support, such as high-flow nasal cannula, prompted several attempts at reclassifying BPD (5, 9). While newer definitions incorporate currently available modalities of respiratory support and are better at predicting longer-term respiratory outcomes, they do not address the different pathogenic mechanisms underlying BPD. There is variability in exposure to pre- and postnatal risk factors as well as variability in NICU practices, some of which injure the airway and parenchymal and vascular compartments differentially, programming several BPD endotypes that affect prognosis, progression, and management strategies (35).

## Current views of BPD pathogenesis and endotypes

### Prenatal risk factors that program the postnatal BPD endotypes.

**Figure 1:**
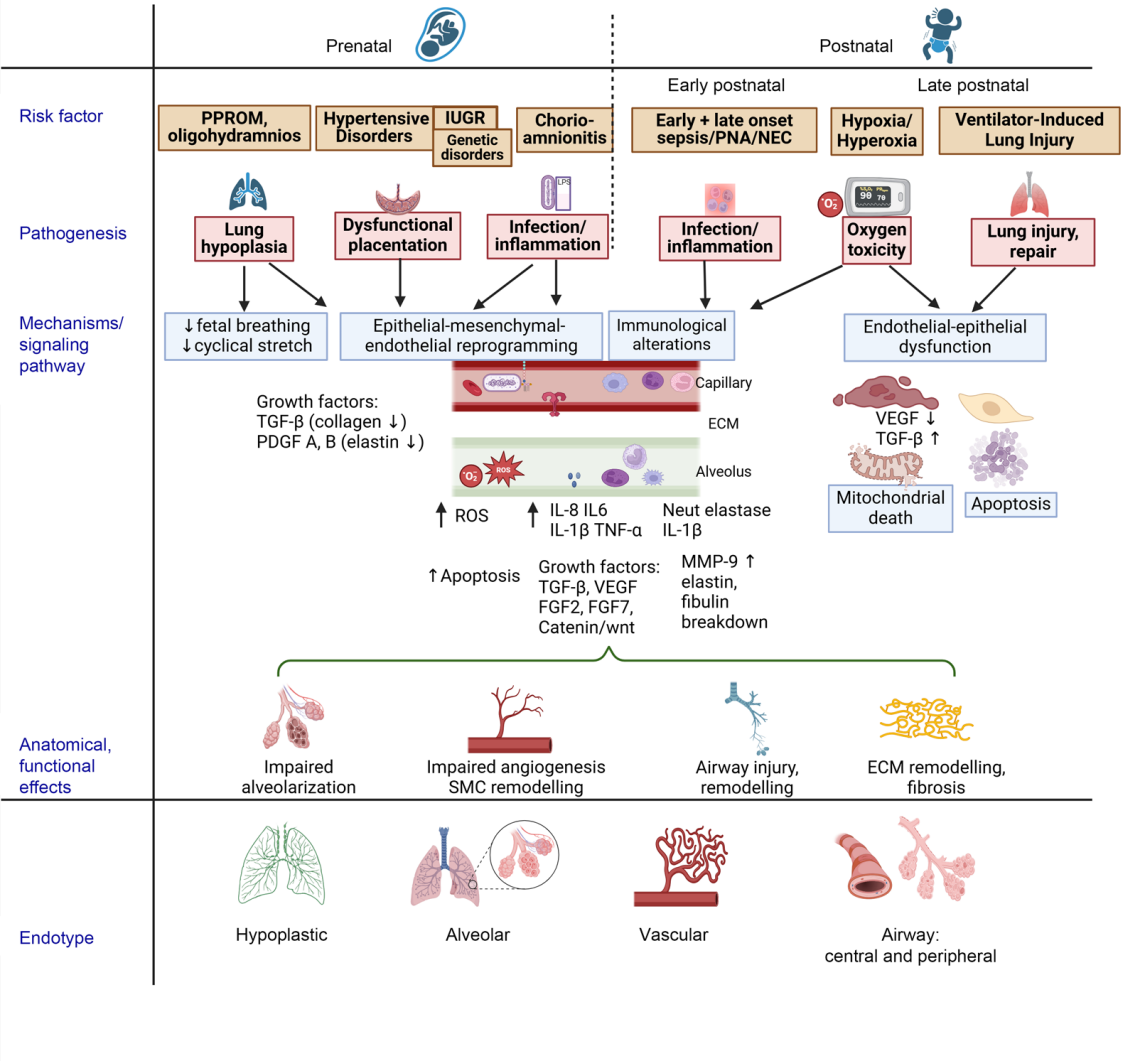
Pathogenesis of BPD showing the links between clinical risk factors, underlying mechanisms, and signaling pathways to disease endotypes. This schematic illustrates how clinical risk factors affect biological mechanisms and signaling pathways in the lung and contribute to the development of BPD endotypes. BPD, bronchopulmonary dysplasia; IUGR, intrauterine growth restriction; PNA, pneumonia; NEC, necrotizing enterocolitis; MMP, matrix metalloproteinases; ECM, extracellular matrix. Created with Biorender.com.

Both prenatal and postnatal injury to different lung compartments, such as the airways, parenchyma, interstitium, and the vasculature, can influence BPD endotypes (Figure 1). As BPD is causally linked to preterm birth, its endotypes are broadly congruent with the three key mechanistic pathways implicated with prematurity that adversely affect the fetal lung.

#### Infection/inflammation.

Infection and/or inflammation underlying chorioamnionitis and preterm premature rupture of membranes (PPROM) are central pathogenic mechanism(s) for prematurity and subsequent BPD. Antenatal exposure to bacterial endotoxins in animal models mimics chorioamnionitis and induces structural abnormalities in the distal lung, contributing to parenchymal and vascular BPD endotypes (36). The inflammatory milieu created by bacteria/ligands (37) can disrupt or reprogram lung morphogenic pathways (38). Prenatal infection/inflammation potentiates dysfunctional alveolar and vascular growth pathways (39), including TGF-β (40), connective tissue growth factor (CTGF) (41), endothelial NOS (42), HIF-1a, Hif-2a (43), VEGF (42), and placental endothelial cell adhesion molecule-1 (42). Endothelial responses (44) to infection/inflammation trigger neutrophil activation (45, 46), with subsequent release of neutrophil elastase (NE), MMPs, proinflammatory cytokines (IL-8, IL-6, IL-1B, TNF), and increased ROS production (47, 48), all of which can remodel the scaffolding of the developing alveoli (49, 50). Additionally, neutrophils induce monocyte infiltration and macrophage activation in the immature lung, thereby initiating a detrimental proinflammatory cytokine cascade that underlies alveolar and airway injury (46). Prenatal exposure to *Ureaplasma* induces a mild but chronic inflammatory response that promotes structural changes in the lung connective tissue and smooth muscle surrounding bronchioles and pulmonary vasculature and modulates the immune system such that it is susceptible to inflammatory stimuli after birth (50–53). *Ureaplasma*-infected lungs, on autopsy, show greater myofibroblast proliferation, interstitial fibrosis, and disordered elastic fibers, corresponding to an interstitial BPD endotype. Clinical findings of cystic emphysematous changes in *Ureaplasma*-infected lungs and of moderate-to-severe small airway obstruction in lung function tests at school age, suggest parenchymal and airway endotypes of BPD (37, 54). Prenatal insults also prime the lung for a second hit after birth upon exposure to other noxious stimuli, such as hyperoxia or mechanical ventilation (55, 56). Several large recent meta-analyses confirmed an association between chorioamnionitis, intrauterine growth restriction (IUGR), and moderate-to-severe BPD (57–59), though significant heterogeneity was noted. A large multicenter cohort study identified histologic chorioamnionitis as a risk factor for grade 2 or 3 BPD and suggested that this association may be primarily mediated through induction of extremely preterm birth (60).

#### Placental dysfunction and fetal growth restriction.

While less well-recognized, hypertensive disorders of pregnancy and IUGR contribute to lung remodeling that predisposes to certain BPD endotypes. Large clinical cohorts have shown that placenta-mediated pregnancy complications, such as gestational hypertension and preeclampsia, with fetal growth restriction associate with moderate-to-severe BPD in very preterm infants (61). Preclinical preeclampsia models reveal that beyond just a small-sized fetus, there is potential for pervasive disruptions in alveolar and lung vascular growth, even in the absence of adverse postnatal exposures (62, 63). Early onset severe preeclampsia is characterized by immunological alterations of the intrauterine milieu, with resultant abnormal placentation and fetal growth restriction (64). There is complex reprogramming of pro- and antiangiogenic factors, oxidative stress, epithelial-mesenchymal transitions, epigenetic alterations, as well as activation of the endoplasmic reticulum stress pathway, all of which remodel the vasculature, mesenchyme, and developing alveoli in utero (62, 65, 66). Preterm infants exposed to placental hypoperfusion and those who went on to develop BPD-associated pulmonary hypertension (BPD-PH) at 36 weeks PMA had decreased cord blood placental growth factor, GM-CSF, and VEGF-A, linking abnormal placentation and ongoing vascular dysfunction to the vascular endotype of BPD (67).

#### PPROM and oligohydramnios.

Recent studies have evaluated the effect of PPROM before 24 weeks gestation on respiratory outcomes (68–70). Distending pressure generated by lung fluid and cyclic stretch of the lung are the two major determinants of fetal lung development. Oligohydramnios is associated with reduced fetal breathing movements, decreased intraluminal distending pressure, and reduced production of stretch-stimulated growth and cell maturation factors and is complicated by additional triggers such as infection/inflammation (71–74). In lamb PPROM models, oligohydramnios reduces lung weight and compliance, while increasing pulmonary vascular resistance (PVR) and reducing pulmonary blood flow (75). Oligohydramnios during the canalicular stage in rats reduced expression of growth factors essential for early branching morphogenesis (TGF-1β for collagen deposition) and alveolarization (PDGF-A and -B with decreased elastin deposition) (71, 74). These preclinical models recapitulate clinical presentation, as preterm infants born after PPROM-associated oligohydramnios exhibit reduced lung volume, ventilatory efficiency, and compliance (76). A recent report from the Spanish registry of preterm infants with BPD provided insight into perinatal risk factors, respiratory support, and spirometry results at early school age (77). Overall, one-third of this entire cohort presented with an airway obstructive pattern, and severe forms of BPD were also associated with a restrictive-mixed pattern, suggesting lung parenchymal injury. Oligohydramnios and chorioamnionitis were associated with obstructive patterns on spirometry. Taken together, pulmonary hypoplasia in the setting of oligohydramnios and PPROM affects both the parenchymal and, perhaps to a greater extent, the vascular components of the respiratory system.

### Postnatal risk factors that program BPD endotypes.

#### Inflammation.

Postnatal lung inflammation, both independently and concurrently with other mediators, commonly results in BPD. Postnatal infectious/inflammatory risk factors may further be classified into early postnatal factors, such as chorioamnionitis, early-onset sepsis, and resuscitation/mechanical ventilation, and late postnatal factors, such as late-onset sepsis, pneumonia/tracheitis, and necrotizing enterocolitis and associated complications. Preclinical studies of postnatal inflammation have often involved treating animals with systemic LPS, which triggers proinflammatory cytokines (IL-1β, IL-6, IFN-γ) and endothelial immune activation through the TLR family of innate immune receptors (78–82). The subsequent recruitment and activation of neutrophils and macrophages causes production of ROS through NADPH oxidase (83), apoptotic endothelial/epithelial damage (84), and microvascular thrombosis (85). Systemic LPS also impairs alveologenesis in a dose-dependent manner by inhibiting expression of growth factors and signaling pathways that regulate branching morphogenesis and alveolar epithelial cell proliferation/differentiation (86–89). LPS administration also results in pulmonary vascular simplification, aberrant angiogenesis and muscularization (through VEGF upregulation), and increased pulmonary arterial pressures, when combined with hyperoxia exposure (90, 91). Proinflammatory activity of neutrophils, via increased neutrophil elastase and myeloperoxidase activity, and macrophagic inflammasome activation (through IL-1β) lead to alveolar oversimplification (86, 87, 92–94) and disruption of the developing elastin meshwork (through MMP-9 activation, ref. 88, and elastin/fibulin degradation, ref. 95) in the distal lung during the saccular stage of lung development. This creates an emphysematous COPD-like airspace dilation with reduced lung function that persists through 24 months of age (92, 95). Lung injury in preterm infants is further propagated by the second hit of pulmonary infections (96). These include *Ureaplasma* colonization/infection of the respiratory tract (97), postnatal CMV infection (98), rhinovirus (99), influenza (100), and respiratory syncytial virus (RSV) infection in preterm infants (101). In summary, multiple studies suggest that postnatal inflammation affects parenchymal, vascular, and airway components by disrupting key morphogenic pathways.

#### Oxidative injury (hyperoxia/hypoxia).

Infants born prematurely have reduced antioxidant defenses and are sensitive to oxidant injury, which can be exacerbated by high concentrations of ambient oxygen (102, 103). Animal models show that exposure of the saccular lung to hyperoxia induces injury to all lung compartments, affecting all three endotypes (104, 105). Exposure of newborn rodents and preterm baboons to ≥85% O_2_ for 10–14 days causes alveolar simplification and abnormal vascular development, analogous to changes seen in preterm infants that die with BPD in the new BPD era (32, 38). Prolonged exposure to hyperoxia results in accumulation of free radicals (H_2_O_2_, superoxide) that initiate inflammatory cytokine release (IL-6, IL-8, IL-1β, IL-1α, TNF-α, monocyte chemotactic protein 1 [MCP-1]), promote epithelial cell apoptosis, and decrease expression of genes required for vascularization (VEGF, TIE2, ANG1) (36, 106–112). Hyperoxia-mediated antagonism of VEGF signaling disrupts postnatal alveolarization and pulmonary vascular growth, and *Vegf* gene therapy can partially preserve and protect the developing rat lung alveolar and vascular components against hyperoxia (113–117). Stunted alveolarization with hyperoxia exposure follows disordered microvascular development, supporting the vascular hypothesis of BPD. Hyperoxia dysregulates Notch 2 signaling and ECM remodeling, leading to abnormal alveolar and distal airway development and lung fibrogenesis (117, 118). Hyperoxia-induced TGF-β signaling disrupts alveologenesis, microvascular development, and causes pulmonary fibrosis (119–122). Adult mice exposed to hyperoxia as newborns exhibit increased airway hyperresponsiveness to methacholine challenge via prolonged and dysregulated immune responses (123). Together, these studies reveal that neonatal exposure to hyperoxia programs airway phenotypes, including airway hyperreactivity and asthma-like symptoms. Hyperoxia in conjunction with other noxious exposures, such as chorioamnionitis and/or LPS, portends worse alveolar and vascular simplification (91) by inducing mitochondrial cell death pathways via apoptosis and necrosis. The pervasive mechanisms by which hyperoxia programs aberrant lung development have been reviewed exhaustively elsewhere (36, 108, 117). In addition to hyperoxia, intermittent hypoxic events with oxygen saturation of <90% increase the risk of BPD and BPD-PH. Whether intermittent hypoxia predominantly induces the vascular endotype remains to be determined (124).

Temporal patterns of fractional inspired oxygen (fiO_2_) during the early postnatal period for extremely low gestational age newborns (ELGANs) can predict BPD severity, and may be indicative of specific BPD endotypes, though these remain uncharacterized (125). Prospective studies in ELGANs have characterized three postnatal oxygen requirement trajectories — a “low fiO_2_” group that maintains <23% fiO_2_ during the first postnatal week, an “early pulmonary deterioration” group that has escalating oxygen (≥25%) needs at day 14, and an “early and persistent pulmonary deterioration” group that requires escalating (≥25%) fiO_2_ throughout the first two postnatal weeks (126). Risk factors for early and persistent pulmonary deterioration include lower gestational age, birth weight, and mechanical ventilation at day 7 but not histological chorioamnionitis or funisitis.

#### Ventilator-induced lung injury.

Mechanisms of ventilator-induced lung injury (VILI) include high airway pressure (barotrauma), large gas volumes in a highly compliant chest wall (volutrauma), alveolar collapse and re-expansion (atelectotrauma), and increased inflammation (barotrauma) (127, 128). Hyperoxia and VILI in neonatal mice causes mitochondrial dysfunction, and the resulting failure of bioenergetics leads to alveolar underdevelopment (129). Alveolar overdistension, through stretch-induced mechanotransduction or shear vascular stress (130), also initiates a proinflammatory response, resulting in increased cytokines expression (IL-1β, IL-6, IL-8, and TNF-α) and altered expression of growth factors (CTGF, CYR61, and EGR1) that are critical for secondary septation and alveolarization (128, 131, 132). Chronically ventilated preterm lambs (>3 weeks) show excessive accumulation of elastin fibers, increased muscularization of terminal bronchioles, inflammation, and defective pulmonary microvascular growth, which inhibits the postnatal decrease in PVR (133, 134). Preterm baboons supported with oxygen and ventilation showed VEGF signaling inhibition, which impaired lung microvasculature development (135). Coalson et al. found that a longer duration (1–2 months) of mechanical ventilation is sufficient to inhibit alveolarization and vascularization (136). Taken together, large-animal studies indicate that mechanical ventilation and oxygen toxicity disrupt parenchymal and vascular development. Similar findings of dysmorphic microvasculature and disordered expression of angiogenic growth factors (Flt-1, TIE-2) have been reported in preterm infants dying with BPD (69). Barotrauma from prolonged positive pressure ventilation of compliant immature airways predisposes the airway to the obstructive endotype of tracheobronchomalacia, though the role of prenatal exposures on major airway injury is less-well studied. Small airway disease can complicate BPD and compound tracheobronchomalacia, as accessory expiratory muscle use increases pleural and transmural pressures generated on the airway walls, worsens airway injury, and contributes to central/peripheral airway malacia BPD endotype (137). Preclinical studies suggest that perinatal risk factors of BPD injure lung compartments differentially programming clinical BPD endotypes.

## Clinical endotypes of BPD based on anatomical and functional characterization

**Figure 2:**
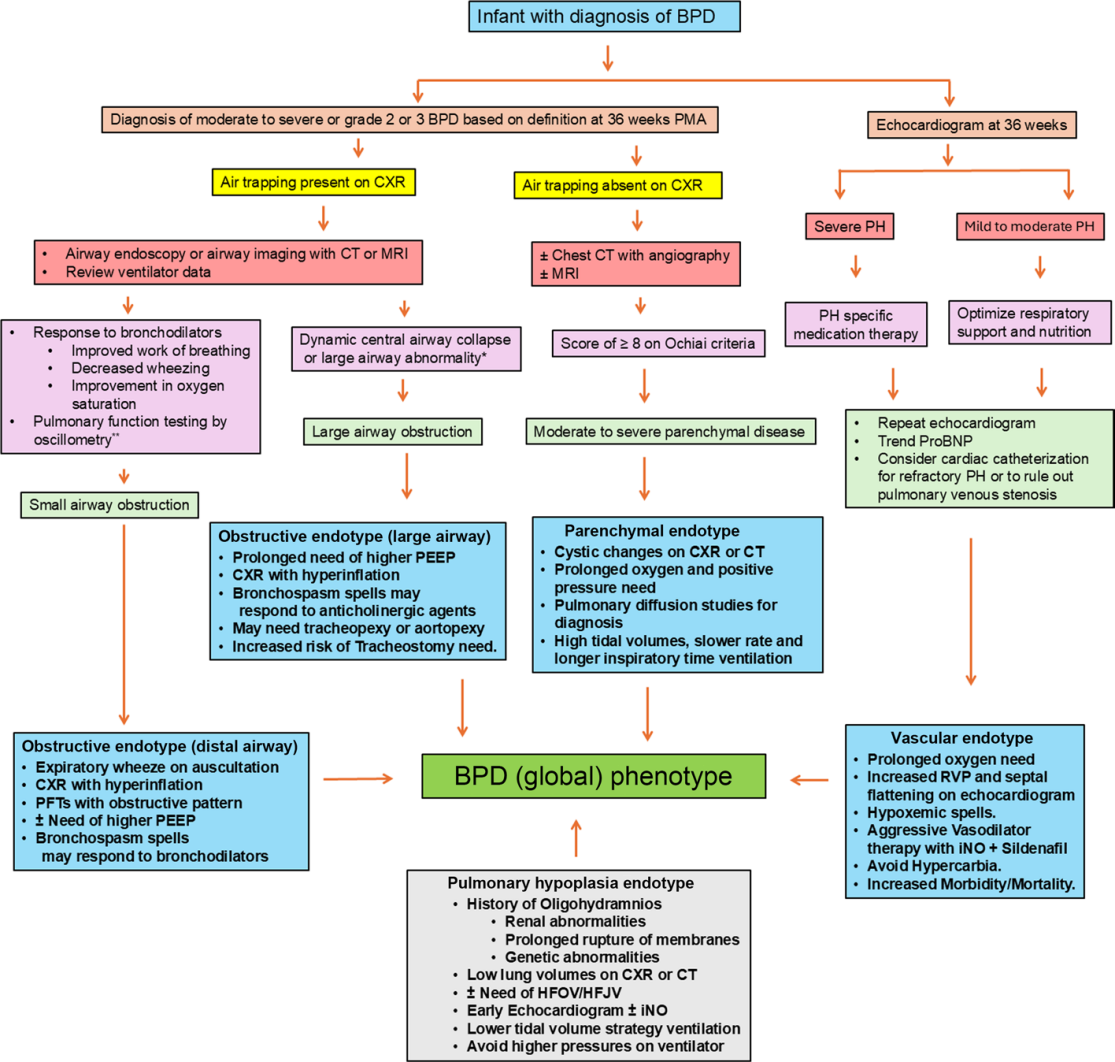
Clinical characteristics, diagnostic evaluation, and targeted treatment approaches for BPD endotypes in premature infants. This figure outlines the clinical features, diagnostic tools, and specialized treatment strategies for BPD endotypes in premature infants. Dynamic airway imaging by CT or MRI may include 3D multidetector computed tomography (MDCT), dynamic 4D MDCT, or ultrashort echo-time magnetic resonance imaging and may be available only in select specialized centers in the United States. Infant pulmonary function testing by oscillometry (either by forced oscillation technique or by impulse oscillometry) is currently available as an investigational tool only. BPD, bronchopulmonary dysplasia; CXR, chest radiograph; CT, computed tomography; MRI, magnetic resonance imaging; PH, pulmonary hypertension; PFTs, pulmonary function tests; ProBNP, pro–brain natriuretic peptide; PEEP, positive end-expiratory pressure; RVP, right ventricular pressure; HFOV, high-frequency oscillatory ventilation; HFJV, high-frequency jet ventilation; iNO, inhaled nitric oxide.

Recent literature suggests that clinical BPD endotypes can be discerned based on clinical symptoms/signs, imaging, and functional testing. Wu et al. used CT with angiography, echocardiography, and bronchoscopy to describe three distinct clinical endotypes of BPD in preterm infants based on the involvement of the lung parenchyma, vascular compartment, and airway (Figure 2). In a cohort of 73 preterm infants with severe BPD, 12% manifested pure parenchymal disease, 8% mainly presented with PH, 7% had isolated large airway disease, and 32% exhibited involvement of all three anatomic components (138). Pierro et al. proposed three additional BPD endotypes, namely, peripheral airway, interstitial, and congestive (10). Peripheral airway disease was diagnosed based on recurrent bronchoconstriction, airway hyperactivity, structural changes, and inflammation in smaller airways in school-age children. An interstitial endotype was proposed based on widening of interstitial spaces and increased fibrosis seen in premature children on lung biopsy, and the congestive endotype was based on increased incidence of pulmonary edema in some children due to insufficient lymphatic drainage (35). BPD classification into distinct endotypes is thus important not only for prognostication but may inform precision targeted therapies (Figure 2).

### Parenchymal endotype of BPD.

The diagnosis of parenchymal disease in BPD is primarily based on serial chest radiographs, CT, and, more recently, MRI techniques, while severity of parenchymal lung disease is determined by the Ochiai scoring system (139), which is a composite of clinical and chest radiography/CT score. The clinical score includes tachypnea, dyspnea, fiO_2_ requirement, pCO_2_ >45 mmHg, and growth rate <25 grams per day. The chest radiograph score is based on evidence of cardiomegaly, hyperexpansion, emphysema, and fibrosis or interstitial abnormalities. The chest CT score is determined by presence of hyperexpansion, intercostal bulging, mosaic pattern of attenuation, blebs, air cysts, thickening of bronchovascular bundle, consolidation, and overall interpretation of BPD severity. The maximum possible total score is 18, and a score of >10 is considered severe parenchymal disease (139). Parenchymal disease management focuses on optimizing respiratory support, reducing VILI, reducing lung inflammation with judicious corticosteroid use, and promoting lung repair by optimizing nutrition and growth. Diuretics are known to decrease airway resistance and improve lung compliance and are used based on the potential for alveolar edema and interstitial lung fluid to complicate BPD. Considering the lack of evidence for long-term benefit and potential for electrolyte imbalances, diuretic use should be limited to a few days (140, 141). Postnatal steroids are commonly used to decrease lung parenchymal inflammation and help facilitate extubation. Several excellent reviews and meta-analyses have addressed their safety and efficacy (142, 143). Dexamethasone targeted to infants with emerging lung disease has been shown to decrease BPD rates, whereas hydrocortisone does not impact BPD rates when used after the first week of life (143).

Radiation exposure from repeated chest radiographs or CT scans increases the risk of leukemia and cancer in children (144, 145); therefore, ultra-short echo-time MRI is becoming a popular radiographic technique to study BPD lung characteristics. Higano et al. compared infants with and without BPD who underwent lung MRI (146) and developed an MRI scoring system based on chest radiograph Ochiai scores. The authors found that BPD severity, respiratory support at the time of discharge, and respiratory support at 40 weeks PMA correlated with the MRI-based scores (146). In addition, Walkup et al. compared the MRI findings of lung parenchyma between preterm infants with BPD and term infants and found that BPD was associated with higher lung volume of signals for atelectasis, edema, and fibrosis (147). Higano et al. reported the potential for inhaled hyperpolarized ^3^He gas diffusion MRI to differentiate alveolar air space size in normal and diseased lungs (148). Other imaging modalities, such as lung ultrasounds, can help clinicians monitor lung aeration and function over time and guide respiratory management. In a recent multicenter prospective study of 147 extremely preterm infants, lung ultrasounds performed at 1, 7, 14, and 28 days of life were able to predict BPD occurrence (149). While these advanced imaging tools are critical for advancing research on functional endotypes, it will take a concerted effort to incorporate these tools into clinical guidelines. Simple bedside functional assessments of shifts in the Sp_O2_/Pi_O2_ curve may help identify parenchymal or vascular endotypes, aiding prognostication (150).

### Vascular endotype of BPD.

The pulmonary vascular endotype may be prenatally programmed, as some newborns continue to have high PVR after birth and have a difficult transition from fetal to neonatal physiology (2). The BPD pulmonary vascular endotype is usually referred to as BPD-PH and is considered late and chronic in the spectrum of pulmonary vascular diseases in preterm infants (151). Clinical indications for screening include infants with established BPD or severe evolving disease and lack of clinical improvement. Echocardiographic parameters used to confirm the PH diagnosis include a tricuspid regurgitation jet velocity of >2.5 ms, estimated right ventricle (RV) pressure >1/2 systemic arterial pressure (moderate) or >2/3 systemic pressure (severe), tricuspid annular posterior systolic excursion *z*-score <–3, pulmonary artery acceleration time <70 ms, RV/left ventricle (RV/LV) end systolic ratio >1.5, LV end systolic eccentricity index >1, and systolic-to-diastolic duration ratio >1.5 (21). A 3D contrast-enhanced lung perfusion MRI has been investigated to assess pulmonary blood flow and lung perfusion, systemic blood flow, cardiac function (including myocardial tissue characteristics), and pulmonary artery–to-aortic (PA/AO) diameter ratios (152). Using cardiac MRI, Crister et al. demonstrated that an increased PA/AO ratio in infants with BPD relates to the severity of the disease. In addition, they found that an increased LV eccentricity index measured by MRI associated with increased hospital stay length, duration of respiratory support, and need for pulmonary vasodilators (20).

Traditionally, a chest CT with angiography is valuable in demonstrating pulmonary vein stenosis, which can cause BPD-PH in infants (153). Due to its invasive nature and risks involved, cardiac catheterization is generally done less frequently and with caution to diagnose PH in infants with BPD. However, for infants with worsening BPD-PH potentially needing a second pulmonary vasodilator, or if pulmonary vein stenosis is suspected, cardiac catheterization should be strongly considered. (21). Acute pulmonary vasoreactivity testing during cardiac catheterization may help predict long-term outcomes of infants with BPD-PH. In a cohort of 26 infants with BPD-PH who underwent cardiac catheterization, 35% demonstrated pulmonary vasoreactivity and had a lower risk of death or tracheostomy compared with nonresponders (154). Pulmonary vein stenosis was associated with a higher mortality but not lung disease severity, as assessed by alveolar-arterial oxygen gradient during cardiac catheterization (155). Optimizing oxygenation via supplemental oxygen, avoiding significant hypercarbia, and use of diuretics and pulmonary vasodilators to prevent RV failure are key to managing the vascular endotype (21).

### Central airway endotype of BPD.

The airway endotype of infants with BPD can be divided broadly into two types based on the anatomic location of airflow obstruction: either the large central airways or the peripheral airway. The central airway endotype primarily manifests as tracheomalacia, bronchomalacia, or subglottic stenosis (156). Bronchoscopy remains the gold standard for the diagnosis of central airway pathologies; however, current diagnostic standards are not universally accepted (157). In addition, bronchoscopy is invasive and requires anesthesia and, thus, is reserved for complex cases. The use of 3D multidetector CT (MDCT) reconstruction images to evaluate large airway abnormalities has been popular in centers with expertise (158, 159). Lee, et al. showed that paired inspiratory-expiratory MDCT can correctly diagnose tracheobronchomalacia in bronchoscopically confirmed cases of tracheobronchomalacia (160). More recently, dynamic 4D MDCT, which combines real-time motion information with anatomic details from 3D volume-rendering CT images, has become a valuable tool in some institutions to evaluate dynamic central airway collapse in infants with BPD (161). The use of higher positive end–expiratory pressures (PEEP) is often required to manage the hyperinflation characteristic of this endotype, and some infants may need tracheostomy for long-term respiratory support (162). Volumetric CT performed with PEEP titrations provides measurements of central airway cross-sectional area and severity of tracheobronchomalacia (163). In 16 infants with severe BPD, PEEP titrations across 4 settings resulted in PEEP optimization that helped the majority, demonstrating that dynamic volumetric CT is a valuable tool for optimizing respiratory support (163).

### Peripheral airway endotype of BPD.

The peripheral airway endotype in BPD consists of airway obstruction, heightened airway responsiveness, and increased susceptibility to wheezing (164). Although this manifestation is similar to asthma, the peripheral airway obstructive pathology in BPD may be due to structural airway remodeling, airway inflammation, and immunoregulatory pathway reprogramming in the preterm lung, rather than being associated with atopy or family history (165). This uncertain etiology may also explain the observed variability in response to bronchodilators and inhaled corticosteroids (58, 164, 166). Several clinical studies have used pulmonary function testing (PFT) in infants with BPD (166–168). However, the lack of appropriate data from infants without BPD limits the interpretation of such measurements. Furthermore, infant PFTs are generally limited to specialized research centers on an investigational basis. Airway endotype management includes use of bronchodilators, secretion clearance to avoid mucus plugging, optimal PEEP to keep airways open, and tracheostomy in severe cases (156, 166, 169, 170).

In a prospective cohort of 110 infants with severe BPD, Shepherd et al. performed infant PFT using raised-volume rapid thoracic compression spirometry and body plethysmography and determined that 51% of these infants had obstructive, 9% had restrictive, and 40% had mixed endotype of BPD (166). Only a subset of infants from all three endotypes responded to bronchodilator therapy, re-enforcing the variability in responses to common BPD treatments. Recently, Nelin et al. reported specific infant pulmonary function test parameters, namely prebronchodilator FEV_0_._5_ and functional residual capacity/total lung capacity ratio, associated with bronchodilator response in infants with BPD (169). Forced oscillation technique (FOT) measures respiratory mechanics by employing small-amplitude oscillations superimposed on normal noninvasive breathing (171). This method reliably assesses bronchial hyperresponsiveness in adults and children. A recent case report illustrated that FOT has potential value in assessing airway obstruction in preterm infants and response to bronchodilator treatment (172). Further studies are ongoing to establish normative FOT data in infants and to develop its application to evaluate peripheral airway disease in infants with BPD (173). Population-based cohort studies among older children with a history of BPD have shown altered airway mechanics and an obstructive pattern in lung function (174). This airway obstructive pathology persists well into adulthood (175).

### Pulmonary hypoplasia endotype of BPD.

Pulmonary hypoplasia in neonates can result from oligohydramnios arising from renal abnormalities or prolonged rupture of membranes; congenital lung malformations, such as congenital diaphragmatic hernia; or genetic conditions that impair lung development (76, 176–178). Specifically, pulmonary hypoplasia should be suspected as a contributor to BPD following PPROM before 20 weeks gestation (177). Autopsies of preterm infants who died of suspected pulmonary hypoplasia reveal smaller lung sizes, volumes, and vascular hypoplasia (76, 177, 178). Management of these infants remains complicated but use of low-volume ventilation strategies and early pulmonary vasodilator therapy might reduce lung injury and optimize outcomes (177). A recent study looked at the effect of oligohydramnios/pulmonary hypoplasia in infants with BPD on childhood pulmonary function and observed significant limitation in pulmonary airflow and structural abnormalities on chest CT scans (179). This study shows that maternal oligohydramnios along with pulmonary hypoplasia significantly affect long-term pulmonary outcomes in infants with BPD, supporting a need for vigilant inpatient management and close outpatient follow-up for infants with a hypoplastic BPD endotype (179).

Characterizing endotypes may help clinicians implement targeted therapeutic strategies that improve long-term outcomes in infants with established BPD. However, given the dynamic evolution of BPD from prenatal priming to postnatal injury, as well as the lack of standardized criteria for both the timing of imaging/investigations and specific interventions, endotype-based management of BPD remains challenging.

## AI for classifying endotypes for BPD

**Figure 3:**
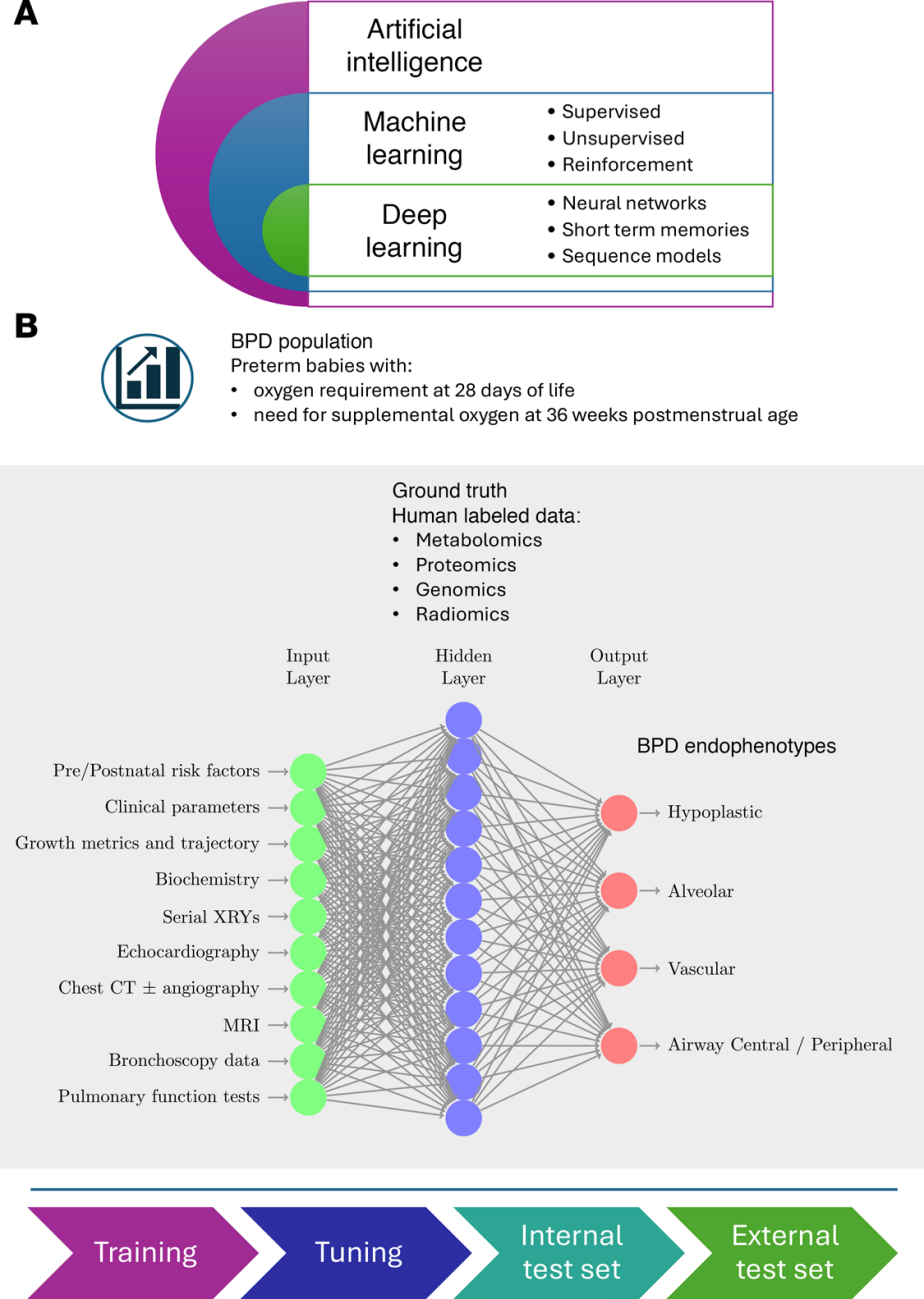
Artificial intelligence for defining and managing BPD endotypes. (**A**) The artificial intelligence (AI) hierarchy demonstrating that deep learning and machine learning are subsets of AI. (**B**) To identify BPD endotypes, a BPD population is identified, followed by supervised labeling of ground truth data. A machine learning algorithm then outputs the relevant BPD endotype. Training and tuning of the machine learning is followed by testing on an internal data and validation on an external cohort.

The use of AI-based approaches to identify and resolve complex patterns hidden in clinical and research data in ways beyond human capacity holds promise for aiding in the classification and endotyping of BPD. Several techniques and approaches are used in AI, including knowledge modeling, symbolic reasoning, and machine learning (ML) (Figure 3). ML can take the form of supervised training models that use human-labeled data to make predictions or classify information. Unsupervised learning discovers hidden patterns and relationships in unlabeled data. Multiomics AI has revealed key pathways, such as those associated with oxidative stress, that can serve to identify BPD endotypes based on the severity of prematurity, genetic risk factors, environmental factors (180), and other antenatal and postnatal variables (181). New multilayered, neural models that integrate biomarkers with clinical data are challenging classic calculators like the National Institute of Child Health and Human Development BPD outcome estimator for prognostication (182, 183).

### ML for early prediction of BPD.

ML algorithms can detect subtle patterns that conventional tools often overlook. Leveraging the Korean neonatal research network, Hwang et al. used prenatal and postnatal clinical variables in a two-stage ML model to predict BPD susceptibility and severity in a large cohort of preterm infants (>16,000) (184). Modeling peripheral blood transcriptomic signatures and clinical variables using ML, Moreira et al. reported that alterations in lung mitophagy, T cell function, alveolar cell renewal, and cell-cell communication genes predicted BPD accurately in the first week of life (183). Verder et al. combined spectral data from gastric aspirates, which provide a measure of the pregnancy lecithin/sphingomyelin ratio, with clinical factors to predict BPD at birth, with high sensitivity and specificity (185). Radiomic data refers to quantitative features extracted from imaging modalities, and AI can be used to analyze these data to characterize endotypes. Using chest radiographs, Chou et al. developed a deep learning model able to predict BPD in preterm infants, showcasing the power of AI to accurately identify radiological features associated with disease severity (186). Combined with MRI to predict anatomical endotypes, AI may help delineate associations among risk variables, anatomical endotypes, and disease progression.

Gilfillan and Bhandari (187) improved on a model developed by Pierro et al. (10) to classify BPD endotypes by incorporating genetic and epigenetic predictors for BPD endotypes. Dai et al. used an ML model that combines exome sequencing data with clinical variables in a cohort of 245 infants, of which 131 had BPD, to demonstrate that genetic factors increased discrimination of the models to predict BPD (188). Genetic studies are particularly important, as BPD risk differs by sex and race (189). The potential for ML modeling to combine multiomics data with clinical data for BPD prediction and endotyping holds substantial promise (190). Multiomic ML prediction models can identify therapeutic windows for early intervention and individualized care by combining biomarkers with clinical risk scores. Finally, by analyzing genetic risk factors for BPD with existing disease/drug databases, promising drug candidates tailored to specific BPD endotypes can be potentially identified (24, 191).

## Challenges and future directions for AI integration into BPD classification

Integration of AI-driven models within a holistic multidisciplinary strategy that incorporates real-time clinical data, respiratory and nutritional trends, and valid omics data remains a challenge (167). The current one-size-fits-all management strategy could be replaced by personalized approaches to BPD endotyping and treatment tailored to each neonate’s genetic signature and disease trajectory. Integration of genomics into model development is limited by the absence of clear genetic risk factors for BPD and limited availability of high-quality data (192), though open-source datasets, synthetic data, and transfer learning techniques could help address this gap (193). Where data are of a high quality, as with radiomic data, BPD endotyping is hampered by the limited use and availability of advanced imaging. CT scans are restricted by radiation concerns, and MRI is cost prohibitive with limited availability. While ultrasound is readily available and increasingly incorporated into AI predictions, it has not been compared with other imaging modalities (192, 194). Ethical considerations surrounding AI, particularly with advanced autonomy, must be addressed through improved communication among AI experts, patients, and ethics specialists (195). AI is noted as an “opaque” system (192) or having “black box” effect (24), referring to a lack of “explainability,” which is crucial for ethical input into AI (195). This lack of transparency can be improved by additional modeling but risks complicating already complex algorithms for clinicians with varying AI understanding (196). Ethical justice is also a concern, as AI models may be trained on nonrepresentative data, challenging equitable healthcare delivery (193). Development of simplified models and scoring systems that incorporate identified variables will facilitate clinician accessibility (24). Specific AI models also have challenges; for example, logistic regression is limited by the requirement that other variables remain constant (197). These considerations affect model selection and highlight the need for AI expertise. Validation is another challenge, as new models should be validated in independent cohorts to strengthen generalizability (183). While integration of AI for BPD prediction and endotyping remains promising, this will require integration of clinical and omics datasets and close collaboration between neonatologists and data scientists. Such coordination will be crucial for advancing the field and bridging the gap between research findings and clinical practice.

## Conclusions

While current definitions of BPD better define disease severity and predict long-term pulmonary outcomes, they do not adequately define pathophysiology-driven endotypes of BPD based on the major lung compartments involved. Such classification is important, as it provides a framework for future precision-based approaches to target developing and established BPD. Better disease definition is more than just a theoretical consideration, as most clinical trials in the last two decades that attempted to prevent the global BPD phenotype had little success (12, 14, 198). Thus, defining BPD endotypes, early in the disease course, will enable endotype-dependent precision therapies to improve outcomes. Defining BPD endotypes is also requisite for optimal management of severe BPD, where tailored approaches are more likely to improve long-term pulmonary outcomes. We speculate that improvements in multiomics technologies and advanced imaging techniques will, aided by AI’s ability to integrate various data streams, result in earlier characterization of BPD endotypes and precision therapies to improve BPD outcomes.

## Author contributions

Both MS and GA contributed equally, as co-first authors, to the conception and writing of this review. MA is listed first because she took the lead in drafting the initial manuscript and integrating revisions.

## Figures and Tables

**Table 1:**
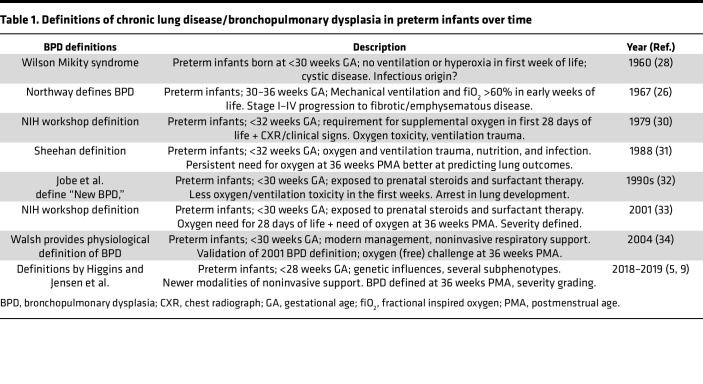
Definitions of chronic lung disease/bronchopulmonary dysplasia in preterm infants over time

## Version 1 10/22/2025

Electronic publication
